# Neural processing of speech sounds at premature and term birth: ERPs and MMR between 32 and 42 weeks of gestation

**DOI:** 10.1016/j.dcn.2024.101444

**Published:** 2024-09-10

**Authors:** Josef Urbanec, Kateřina Chládková, Jan Kremláček

**Affiliations:** a Department of Medical Biophysics, Faculty of Medicine in Hradec Králové, Charles University, Czechia; b Institute of Czech Language and Theory of Communication, Faculty of Arts, Charles University, Prague, Czechia; c Institute of Psychology, Czech Academy of Sciences, Prague, Czechia

**Keywords:** Newborn speech perception, Event-related potentials, Mismatch response, Perinatal development, Premature birth, Vowels

## Abstract

Prenatal listening experience reportedly modulates how humans process speech at birth, but little is known about how speech perception develops throughout the perinatal period. The present experiment assessed the neural event-related potentials (ERP) and mismatch responses (MMR) to native vowels in 99 neonates born between 32 and 42 weeks of gestation. The vowels elicited reliable ERPs in newborns whose gestational age at time of experiment was at least 36 weeks and 1 day (36 + 1). The ERPs reflected spectral distinctions between vowel onsets from age 36 weeks + 6 days and durational distinctions at vowel offsets from age 37 weeks + 6 days. Starting at age 40 + 4, there was evidence of neural discrimination of vowel length, indexed by a negative MMR response. The present findings extend our understanding of the earliest stages of speech perception development in that they pinpoint the ages at which the cortex reliably responds to the phonetic characteristics of individual speech sounds and discriminates a native phoneme contrast. The age at which the brain reliably differentiates vowel onsets coincides with what is considered term age in many countries (37 weeks + 0 days of gestational age). Future studies should investigate to what extent the perinatal maturation of the cortical responses to speech sounds is modulated by the ambient language.

## 1 Introduction

The attunement to the native language begins during the last period of intrauterine development, sometime after the 28th week of gestation when the auditory pathways are in place. Near-term fetuses and newborn infants recognize their mother's voice, the global characteristics of the language their mother spoke during pregnancy, as well as rhymes she recited during the last weeks of pregnancy (DeCasper and Fifer, 1980, May et al., 2018, DeCasper et al., 1994). The prenatal learning of spoken language is more intricate than pure remembering of global language patterns: there is evidence that by the time they are born humans have already started to generalise over the linguistic structures such as the intonational and rhythmic patterns specific to their native language (Mampe et al., 2009, Abboub et al., 2016). Moreover, studies indicate that the prenatal learning of native-language patterns might pertain even to smaller-sized structures such as the identities of individual vowels and syllables (Moon et al., 2013, Partanen et al., 2013, Chládková et al., 2021). While current behavioural and neuroimaging literature demonstrates that humans do have the various language-specific abilities at the time of birth, it still remains unknown when exactly during prenatal development the attunement to native linguistic patterns sets on. The aim of our experiment is to advance the current understanding of the very beginnings of spoken language development by testing at what gestational age the newborn cortex distinguishes between minimally contrastive native-language syllables.

The literature indicates that the ability to discriminate syllables develops sometime between the 28th and 35th week of gestation: fetuses stimulated with syllables [ba] and [bi] show behavioural signs of discriminating such stimuli at the 35th but not at the 28th week of gestational age (Lecanuet et al., 1987). Using an indirect measure of neural activity, the brain's hemodynamic response, Mahmoudzadeh et al. (2013) tested discrimination of consonant-vowel syllables in twelve preterm infants' born between 28 and 32 weeks of gestational age. Different patterns of hemodynamic activity were detected for stimulation with strings of repeating identical syllables [ga] compared to strings of oddball blocks with two different syllable identities [ga] and [ba]. Using data from the same experimental session, Mahmoudzadeh et al. (2017) measured the ERPs and reported neural discrimination of the (predictably occurring) changes in syllable identity (as well as speaker voice). The results suggested that the cortex of preterm newborns distinguishes between the two different syllables. Daneshvarfard et al. (2019) assessed the frequency following response (FFR) in the cortical auditory responses to strings of [ba] and [ga] in 16 preterm newborns born between 29 and 34 weeks of gestation. They found that the accuracy and the phase coherence of the response correlates with age, suggesting development of the frequency-following response across the tested preterm age range.

The period between approximately the 30th and 36th week of gestation reportedly marks a change in the cortical as well as subcortical processing of sounds. Starr et al. (1977) examined the auditory brainstem responses (ABRs) to nonspeech stimuli (clicks) in 42 newborn infants ranging in age between 25 and 44 weeks of gestation, and found that the ABRs stabilise in gestational week 36. It is in the same period when also the cortical responses, the auditory event-related potentials (ERPs) of prematurely-born neonates change in their appearance and come to resemble those of fullterm newborns. This change in the ERPs is characterised by a shift from a dominant negative peak to a dominant positive peak at the latency of about 200–250 ms after the onset of an auditory (non-speech) stimulus (Rotteveel et al., 1987; Eggermont and Moore, 2012). The auditory event-related potentials change significantly from birth up until adolescence. The auditory ERP waveforms in infants born extremely preterm at 24 weeks display a negative peak at about 200 ms post stimulus onset and a positive peak at about 600 ms, whose latency decreases with development. At term, it is the positive peak that comes to dominate the auditory cortical response with a latency of about 250 ms post stimulus onset while the negative component seen in extremely premature infants is no longer visible in the ERP waveform (Eggermont and Moore, 2012). This dominance of the large positive peak at about 200 ms latency remains a characteristic of infant and toddler auditory ERP for at least several years; the negative N1 component, characteristic of auditory ERP in adults, fully develops only at about 5–6 years of age or even later (Lippé et al., 2009, Ruhnau et al., 2011). The maturation of auditory ERPs is observed earlier at midline regions and later also at temporal sites (Guzzetta et al., 2011). The degree of ERP maturation is affected by the mode of stimulus presentation: an adult-like N1 can be observed at younger ages with longer inter-stimulus intervals and at older ages with shorter inter-stimulus intervals (Ruhnau et al., 2011).

While there are a number of studies that assessed the cortical processing of speech stimuli between preterm and fullterm newborns, they do not allow to make inferences about the developmental trajectory of cortical speech sound processing because comparisons were made between fullterm infants and preterm infants at term age (François et al., 2021, Pena et al., 2012; Kostilainen et al., 2020). In order to pinpoint the age at which discrimination of native speech sounds starts to be reliably indexed by the auditory event-related brain potentials, our experiment assesses the event-related potentials in 99 newborns spanning gestational ages 32–42 weeks who were all tested within a few days after birth.

As to stimulus characteristics, prior research shows that newborns' brains process speech and nonspeech stimuli differently (when presented with continuous speech, May et al., 2018, but also when presented with isolated syllables, Chládková et al., 2021), one can thus expect that the developmental trajectory of auditory ERPs will differ between speech and nonspeech stimuli. Here we focus on the development of cortical processing specific to speech, which is modulated not only by auditory and neural maturation but also by prenatal speech input, and which may very likely differ from the development of cortical processing of non-speech sounds. The present experiment aims to show when in gestational development the cortex discriminates between minimally distinct speech sounds of the ambient language.

The maturational stages of auditory ERPs have been relatively well documented for changes *between* infancy, toddlerhood, childhood, and adolescence as well as within adulthood (Wunderlich et al., 2006; Ruhnau et al., 2011; Mahajan and McArthur, 2012; Tomé et al., 2015) but are considerably less well documented *within* infancy or *within* gestational development as such (Kushnerenko et al., 2002). Given that auditory ERPs to speech at birth have been repeatedly shown to correlate with later language outcomes and language-related disorders both in full-term and in premature infants (Thiede et al., 2019, Maitre et al., 2013), it is necessary to have a more detailed understanding of how the cortical auditory processing develops in the earliest stages of development, and particularly so for speech sounds.

While it may take several years for the maturation for the primary auditory ERP components such as the N1 and P2 to complete, studies with young infants often focused on a secondary ERP measure, the mismatch response (MMR), as an index of auditory development, and speech perception development in particular. The MMR is assessed in a difference waveform obtained by subtracting the ERP to one type of stimulus (a frequently presented one) from an ERP to another type of stimulus (an infrequently presented one). While some consider MMR an ontogenically early ERP response (Stefanics et al., 2007) others underline its status as being an investigator-developed construct as it is never measured directly from the scalp (unlike the N1 or P2 components) but only obtained through subtraction of the recorded ERPs (Eggermont and Moore, 2012). Despite that, studies on auditory and speech processing with young infants or even fetuses largely rely on the MMR. Considering the MMR as an index of maturation might not be straightforward: it turns out that to reliably identify which factors affect the MMR polarity and latency in infants is not trivial, and at the same time, it becomes clear that age alone is not the primary modulating factor (Govaart et al., 2023). Interpreting the MMR with reference to the primary ERP responses thus allows to more comprehensively assess the development of early cortical processing of speech. To investigate how the developing cortex responds to different native speech sounds, we thus measure the primary auditory ERPs. To investigate phonetic discrimination beyond the primary sensory processing of acoustic stimulus differences, we measure the neural discrimination index, the MMR.

We presented sleeping newborns with trains of isolated vowels from their native language, Czech, which differed in spectral quality or in duration. As acoustic signals pass through the maternal tissue, abdomen, and bones, their spectral properties from about ∼ 1000 Hz and above are attenuated while durational properties are transmitted veridically (Richards et al., 1992; Granier-Deferre et al., 2011). One can assume that if the ambient language systematically differentiates vowels not only in terms of spectral properties but also in terms of duration by having short and long vowel categories (as Czech does), the developing fetus may more robustly sensitise to speech sound contrasts cued by duration. We thus predicted that Czech-exposed newborns may begin to differentiate differences in vowel duration earlier than vowel spectral properties, which might be indexed by a more mature MMR response and/or differences in the primary ERP responses. However, considering that vowel duration is cued at stimulus offset, and vowel spectral quality at stimulus onset, a confound comes to play whereby offset ERPs are reported to be in general weaker than onset ERPs (in adults, Baltzell and Billings, 2014). To this end, the MMR will provide valuable insights into the neural discrimination of durational vs spectral vowel contrasts as it is not dependent solely on stimulus physical properties (unlike the ERPs) but also on the auditory system abstracting away from the immediate stimulus, building up predictions on the upcoming vowel identity and evaluating violations to those predictions (Garrido et al., 2009). Besides allowing us to trace the early development of cortical responses to speech sounds, the present experiment will enable us to compare the developmental trajectory across different types of speech stimuli.

The present study assesses sensory cortical processing of different native vowels as well as the neural index of phonetic discrimination. Tracing the brain's speech sound processing across neonate infants born between 32 and 42 weeks of gestation age will allow us to identify the ages at which the cortex reliably distinguishes across native speech sounds, and compare the maturation of speech-elicited ERPs to prior findings on perinatal auditory processing of nonspeech signals.

## 2 Method

### 2.1 Participants

**Fig. 1:**
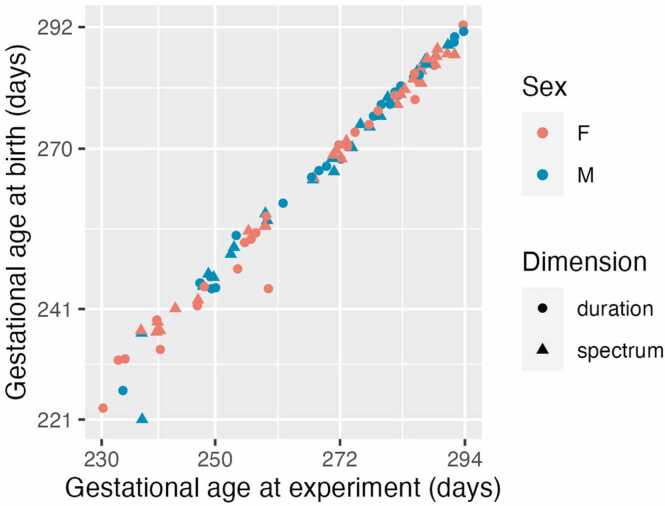
The 99 participants in the present experiment. Points show individual participants; x axis = gestational age at time of experiment; y axis = gestational age at birth; the distribution of females and males in colour; and assignment to stimulation groups indicated by different shapes. Dimension was a between-subject factor, dividing our total sample into two groups of n = 51, and n = 48, respectively, for the stimulation with the spectral contrast and for the stimulation with the durational contrast. Gestational age in days was modelled as a continuous factor.

A total of 102 infants were tested, 3 of them were excluded due to administration of unusual neonatal drugs, congenital malformation of the brain and cardiopulmonal resuscitation after delivery. Data of 99 infants were retained for analysis. Fig. 1 shows their gestational age at birth and at time of experiment, sex, and the condition to which they were (randomly) assigned. The infants were born between the 32nd and 42nd gestation weeks and tested on the 3rd day after birth (range 1–16 days, in the most preterm babies usually in the second week of life because of previous life support). Their birth weight ranged from 1500 g to 4370 g. All infants had 10-min Apgar scores 8 or higher and passed the neonatal hearing test (typically administered the 3rd day after birth in fullterm newborns). Newborns delivered by vacuum extraction or forceps were not recruited. The infants were born to women whose native language was Czech. The experiment was approved by the ethics committee of Havlíčkův Brod hospital, Czechia. Infants took part in the experiment following a parental written informed consent.

### 2.2 Stimuli and paradigm

**Fig. 2:**
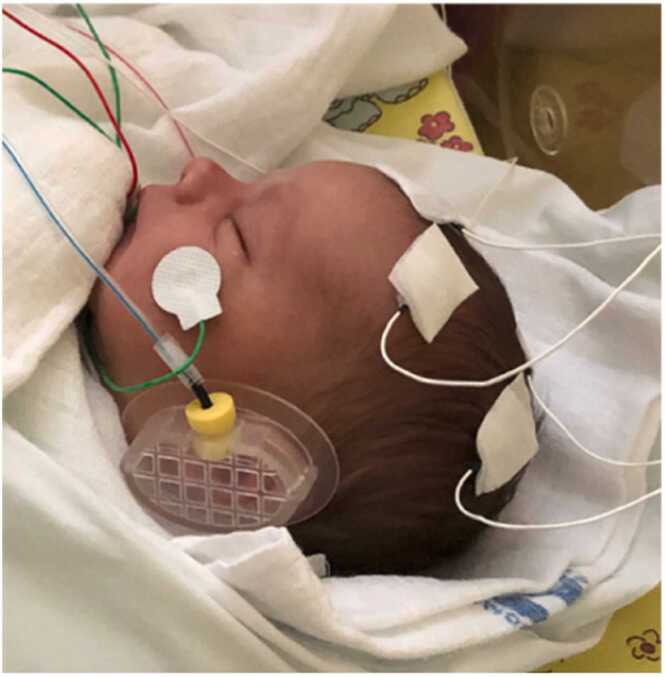
An asleep infant taking part in the experiment. The photo displays 2 of the 6 EEG sensors attached on the scalp (here, at locations F3 and C3), an external channel on the face (grounding), and one of the infant ear couplers with an insert earphone (the online-reference channel on the right side of the nose is not visible in this figure).

Infants were assigned to one of two conditions, receiving either durational-change or spectral-change stimulation. The durational condition tested the contrast between [ɛ] and [ɛː] and the spectral change condition tested the contrast between [ɛ] and [a], both vowel contrasts representing a phonemic change in Czech, the infants' native language. The vowels were from natural recordings of a Czech female speaker who produced a series of [f]-vowel monosyllables. For each vowel category, the most clear and prototypically-sounding vowel was extracted as the middle 50 % portion of the vocalic interval and edited for duration using PSOLA in Praat (Boersma and Weenink, 1992–2024). The first three formant values of [ɛ] were 755 Hz, 1646 Hz, and 2710 Hz, and the first three formant values of [a] were 864 Hz, 1287 Hz, and 2831 Hz. The duration of the short [ɛ] and [a] was 180 ms, and the duration of the long [ɛː] was 360 ms. The stimuli were presented at 65 dB SPL via insert earphones attached on the inner side of infant ear couplers. Fig. 2 illustrates the setup.

Each infant listened to two oddball blocks in which the standard and the deviant swapped roles. That is, for the spectral condition, one of the blocks had [ɛ] as standards and [a] as deviants, and vice versa for the other block, with the order of blocks being counterbalanced. The durational change blocks were analogous, one block with [ɛ] as standard and [ɛː] as deviant, the other with the role of the two vowels reversed. Each block contained a total of 843 stimuli out of which 120 were deviants (deviant probability being 14.2 %). A block always started with 9 standards and subsequently there were 3–9 standards between successive deviants. The stimulus onset asynchrony jittered randomly between 990 ms and 1190 ms (in 10-ms steps). Each block lasted 15.3 min. There was a brief break between the blocks to allow switching stimulation and checking electrode impedances with the infant kept asleep.

### 2.3 EEG recording and procedure

EEG was recorded from 6 scalp electrodes placed at the locations F3, Fz, F4, C3, Cz, and C4 according to the international 10/20 system. External electrodes were placed on the nose (online reference), on the face (grounding electrode), and on the chest or a hand to monitor ECG. EEG was recorded at a 1000-Hz sampling frequency. Impedances were kept below 50 kΩ. Infants were tested while asleep; infant state was monitored by a video camera. The experimenter (the first author) and in most cases also the infant's mother were silently present in the testing room during the whole recording session. Fig. 2 shows the recording setup in one of the infants.

### 2.4 EEG preprocessing

The signal amplifier's bandwidth spanned from 0.3 to 100 Hz (DEYMED Diagnostic s.r.o., Czech Republic). Data processing was carried out using Matlab release 2023a (Mathworks, USA). Frequencies exceeding 40.0 Hz in the recorded EEG were eliminated using a digital filter (using the inverse Fast Fourier Transformation, implemented in EEGLab as eegfiltfft, Delorme and Makeig, 2004). As a result, the spectral composition of the analyzed EEG was constrained to 0.3–40.0 Hz. The EEG signal underwent epoching, commencing 100 ms before and concluding 1000 ms after the vowel onset. The average voltage of the prestimulus segment (from − 100 ms to 0 ms) was subtracted from each epoch. Individual ERPs were computed by averaging epochs in which the absolute amplitude at any sample was below 90 μV, at any electrode site. This procedure led to the rejection of approximately 39 % of epochs (the rejected artefacts were mainly due to movement of the sleeping newborns and the associated slight shifts in the position of the electrodes, which can modulate the polarisation voltage, leading to changes in the recorded signals; some artefacts were probably also due to transitions between sleep stages, eye and involuntary muscle movements in active sleep). Table 1 displays the mean number and range of retained epochs, aggregated across infants and channels. Furthermore, the ERPs were subjected to offline digital filtering using a low-pass Savitzky-Golay filter (Press et al., 1992) with a first polynomial order and a window of 21 samples. This filtering enhanced the legibility of the responses.

**Table 1:** The average, the minimum, and the maximum number of epochs, pooled across infants (divided in two age bins for the purpose of the artefact rejection statistics in this Table only) and channels, for each stimulus type. The row for "ɛ(ɛ:)" represents the number of epochs for [ɛ] that were presented in the same session with [ɛ:] Analogously, the row for "ɛ(a)" represents the number of distinct epochs of [ɛ] played in the same sessions with [a].

		ERP standard			ERP deviant		
Age bin	Stimulus	min	mean	max	min	mean	max
Fullterm	ɛː	89	291	438	17	71	119
	ɛ (ɛː)	96	288	475	23	71	110
	ɛ (a)	61	311	473	18	75	114
	a	86	297	474	11	76	116
Preterm	ɛː	90	288	461	20	77	112
	ɛ (ɛː)	73	311	443	30	71	116
	ɛ (a)	126	271	440	26	68	111
	a	86	293	435	27	62	109

### 2.5 Statistical analysis

Onset ERPs and offset ERPs were computed for standard stimuli in the spectral and duration conditions, respectively, excluding the two standards immediately following a deviant. Onset ERP was quantified as the area under curve in a window between 150 ms and 400 ms after vowel onset: in order to assess the ERP response related to the spectral difference that sets on at vowel onset, the window between 150 ms and 400 ms was intended to capture first ERP peak that in young (incl. premature) infants reportedly has a latency of about 200–250 ms and is considerably wider than adult ERPs (Eggermont and Moore, 2012). Offset ERP was quantified as the area under curve in a window between 400 and 650 ms after vowel onset: in order to quantify the ERP response related to the durational difference between the short and the long vowel, offset ERP was assessed in a 250-ms window starting 220 ms after the offset of the short vowel.

Difference waves were calculated for physically identical stimuli, whereby the ERPs to standards from one block were subtracted from the ERPs to deviants – physically identical stimuli as the standards – from another block. MMR was calculated as area under curve in two time windows of the difference wave: an early window 80–220 ms after change onset and a late window 500–700 ms after change onset; the change onset coincided with vowel onset for the spectral change between [ɛ] and [a], and with the end of the short vowel for the duration change between [ɛ] and [ɛː].

Onset ERPs, offset ERPs, and the MMR were analyzed with linear mixed-effects models. The analysis for onset ERP modelled Age (i.e. gestational age at time of experiment, continuous numeric factor, centered to 259 days, i.e. 37 weeks, considered as the threshold of term age), Stimulus (*a* vs *e*, coded as − 1 vs + 1), Region (lateral sites F3, C3, F4, C4 vs midline sites Fz, Cz, as − 1 vs + 1), and their interactions, and Sex (female vs male, coded as − 1 vs + 1) as fixed factors, and per-participant intercept and slopes for Stimulus and Region as random factors. The analysis for offset ERP modelled Age (numeric, centered to 259 days), Stimulus (*long ee* vs *short e*, coded as − 1 vs + 1), Region (lateral sites F3, C3, F4, C4 vs midline sites Fz, Cz, as − 1 vs + 1), and their interactions, and Sex (female vs male, coded as − 1 vs + 1) as fixed factors, and per-participant intercept and slopes for Stimulus and Region as random factors. The model for MMR included Age (continuous numeric factor, centered to 259 days), Contrast (durational vs spectral, coded as − 1 vs + 1), Window of analysis (early vs late, coded as − 1 vs + 1), Direction of change (a change from [ɛ] to [ɛː] or [a] coded as − 1, vs a change towards [ɛ] from [ɛː] or [a] coded as + 1), and Region (lateral vs midline, coded − 1 vs + 1) as well as their interactions, and a main effect of Sex (F vs M), as fixed factors, and a per-participant random intercept. The models were run in R (R Core team, 2022) using the packages *lmer* and *lmerTest* (Bates et al., 2015, Kuznetsova et al., 2017), means were estimated with *ggeffects* (Lüdecke, 2018).

## 3 Results

### 3.1 ERP results

**Fig. 3:**
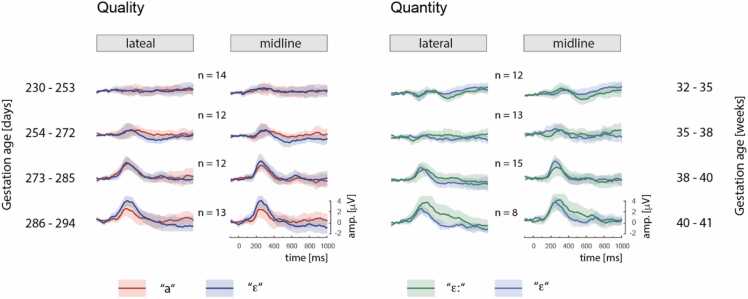
ERPs to standards in infants tested with the spectral change between [a] and [ɛ] (left) and in infants tested with the durational change between [ɛː] and [ɛ] (right). Individual rows show responses averaged across infants in one of four age bins (the age bins are used only for visualisation; analyses were done with age as continuous factor). The figure shows averages for the lateral sites (F3, C3, F4, C4) and the midline sites (Fz, Cz). Shaded areas represent 95 % confidence intervals of the mean ERP waveforms.

Fig. 3 plots the ERP waveform to Standard stimuli in each condition. The ERPs in the Spectral condition were statistically analyzed with the Onset models, and the ERPs in the Duration condition with the Offset models. The fixed-effects model summaries for Onset and Offset ERPs are shown in Table 2 and Table 3, respectively.

**Table 2:** Fixed-effects model output for onset ERP.

Parameters for ONSET ERP	Estimate	Std. Error	df	t value	Pr(> |t|)
(Intercept)	201.315	52.07	49.413	3.866	< 0.001
Age (mean-centred)	12.097	2.592	49.36	4.668	< 0.001
Stimulus (-a + ɛ)	− 40.6	18.788	560.98	− 2.161	0.031
Region (-lateral + midline)	37.32	18.784	560.935	1.987	0.047
Sex (-F + M)	69.728	45.673	47.925	1.527	0.133
Age:Stimulus	− 1.917	0.934	561.191	− 2.051	0.041
Age:Region	− 2.122	0.933	560.935	− 2.274	0.023
Stimulus:Region	− 18.449	18.784	560.935	− 0.982	0.326
Age:Stimulus:Region	0.289	0.933	560.935	0.309	0.757

**Table 3:** Fixed-effects model output for offset ERP.

Parameters for OFFSET ERP	Estimate	Std. Error	df	t value	Pr(> |t|)
(Intercept)	− 19.849	42.427	47.656	− 0.468	0.642
Age (mean-centred)	2.111	2.116	47.588	0.998	0.324
Stimulus (-e:+ e)	15.628	21.412	522.000	0.730	0.466
Region (-lateral + midline)	− 5.680	21.412	522.000	− 0.265	0.791
Sex (-F + M)	− 46.455	38.621	45.000	− 1.203	0.235
Age:Stimulus	3.859	1.055	522.000	3.659	< 0.001
Age:Region	0.340	1.055	522.000	0.322	0.747
Stimulus:Region	− 4.868	21.412	522.000	− 0.227	0.820
Age:Stimulus:Region	− 1.225	1.055	522.000	− 1.161	0.246

**Fig. 4:**
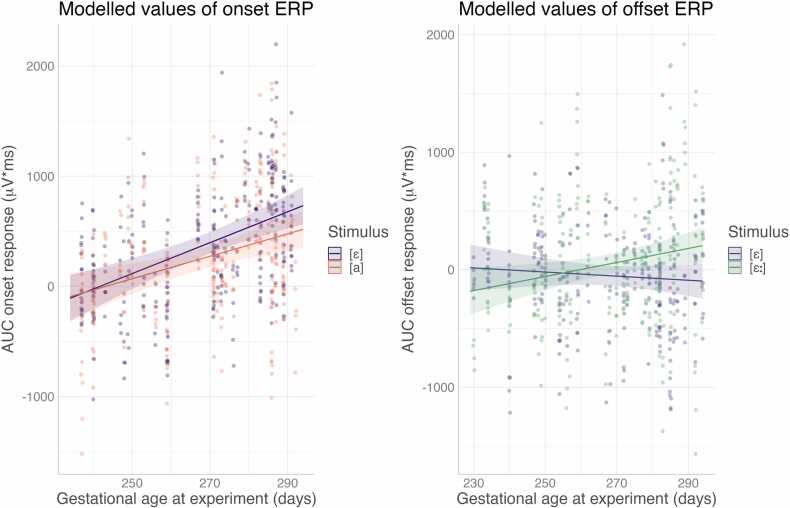
Modelled amplitude of the Onset ERP to standards in the spectral condition (left) and to Offset ERP to standards in the duration condition (right). The thick curves show estimated means and shaded sleeves represent 95 % confidence intervals; points show raw data.

In the model for Onset ERP, the significant intercept indicates that overall there was an onset response reliably different from 0, with mean area under curve estimated at 201 μV·ms. There was also a main effect of Age, showing that the higher the age the larger the peak. Inspection of the estimated means shows that the onset ERP amplitude was reliably larger than 0 from day 253 of age (gestational age at time of experiment). There were also significant main effects of Stimulus and Region, suggesting that the Onset response was larger for the standard [ɛ] than for the standard [a], and larger on the midline than laterally. There were also significant two-way interactions of Age and Stimulus, and of Age and Region. The interaction of Age and Stimulus is directly relevant to our research question: "At what age does the ERPs reflect differential processing of different native vowels?". The interaction is visualised in Fig. 4 (left), which indicates that the Onset ERPs to [a] and the Onset ERPs to [ɛ] start to differ from one another with increasing age. Inspections of the estimated means across the age range show that the onset ERPs to [a] and [ɛ] differ reliably from the 258th day of gestational age at time of experiment (pooled across the midline and lateral regions).

The model for Offset ERP did not yield a significant intercept, suggesting that overall no reliable Offset response was detected across conditions. There was a significant interaction of Age and Stimulus. As shown in Fig. 4 (right), the Offset ERPs to [ɛː] and the Offset ERPs to [ɛ] start to differ from one another with increasing age. Inspections of the estimated means across the age range show that the Offset ERPs to [ɛː] and [ɛ] differ reliably from the 265th day of age (pooled across the midline and lateral regions).

### 3.2 MMR results

**Fig. 5:**
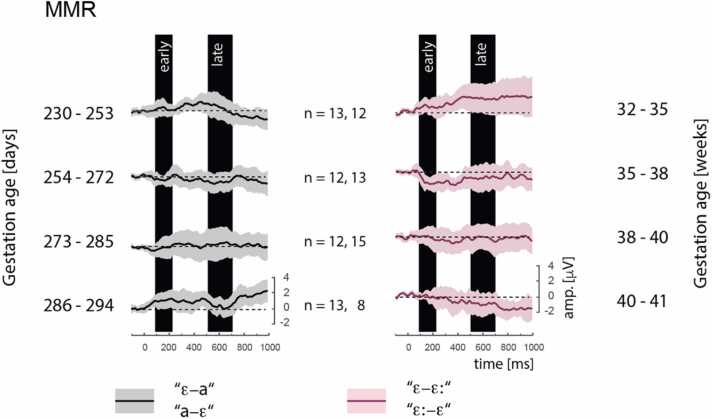
Left: Difference waves for the spectral contrast (*ɛ-a or a-ɛ*). Right: Difference waves for the durational contrast (*ɛ-ɛ: or ɛ:-ɛ)*. Individual rows show grand-average difference waves in four age categories averaged over all recorded leads (F3, C3, F4, C4, CZ, FZ); the figure pools across both directions of change within each contrast type (for each direction of change differences waveforms were computed from physically-identical stimuli from different blocks and then averaged across the two directions of change). The black bars depict the time intervals used for the early (180–220 ms) and the late (500–700 ms) analysis window to compute the area under curve (AUC). Shaded areas represent 95 % confidence intervals of the mean difference waveforms.

**Fig. 6:**
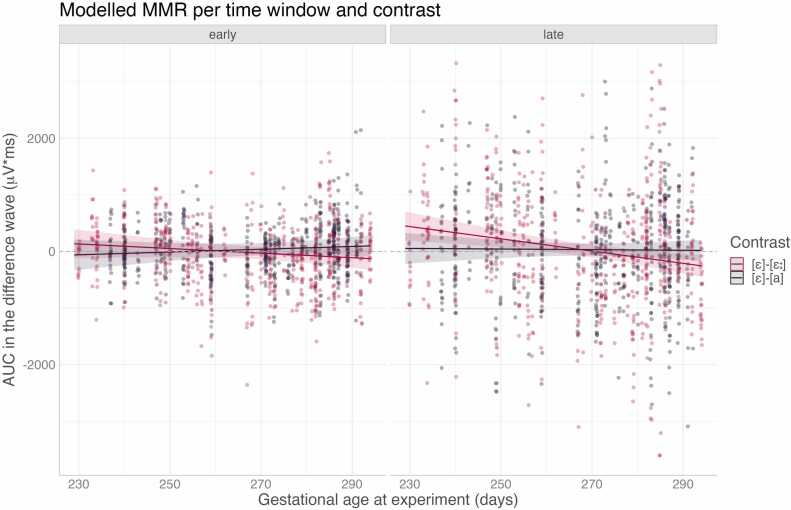
Modelled MMR per time window and per contrast, i.e. the durational (red) and the spectral change (black). Thick curves show estimated mean and shaded sleeves 95 % confidence intervals; points show raw data.

The difference waveforms are shown in Fig. 5. The fixed-effects model summary for MMR is shown in Table 4. As per the non-significant intercept, the analyses found no evidence of a reliable MMR across ages and conditions. However, Age was found to interact with the Window of analysis and with Contrast (Age:Latency: mean slope = − 2.443, *p* = 0.010; Age:Dimension: mean slope = 4.197, *p* = 0.036). Fig. 6 plots the MMR amplitude across the age range separately for each contrast and each window. It can be seen that the MMR amplitude gets more negative (supposedly indicating a more mature response) with increasing age, and especially so for the durational [ɛː]-[ɛ] contrast in the late MMR window. Inspection of the estimated means and their confidence intervals shows that a reliable MMR response is detected (only) for the late MMR to [ɛː]-[ɛ], which has a positive amplitude at the younger ages and becomes reliably negative (95 % conf.int. below zero) at the age of 285 days.

**Table 4:** Fixed-effects model output for MMR.

Parameters for MMR	Estimate	Std. Error	df	t value	Pr(> |t|)
(Intercept)	47.880	40.397	108.284	1.185	0.239
Age (mean-centred)	− 3.241	1.991	104.439	− 1.628	0.106
Dimension (-duration + spectrum)	− 22.736	39.834	104.867	− 0.571	0.569
MMR latency (-early + late)	34.155	19.257	2252.886	1.774	0.076
Direction of change (-from /E/, + to /E/)	17.255	19.258	2252.933	0.896	0.370
Region (-lateral + midline)	15.291	15.674	2252.886	0.976	0.329
Sex (-F + M)	37.877	34.746	92.868	1.090	0.276
Age:Dimension	4.197	1.979	104.504	2.120	0.036
Age:Latency	− 2.443	0.952	2252.886	− 2.565	0.010
Dimension:Latency	− 21.286	19.257	2252.886	− 1.105	0.269
Age:Direction	0.461	0.952	2253.205	0.484	0.628
Dimension:Direction	− 23.118	19.258	2252.933	− 1.200	0.230
Latency:Direction	− 2.621	19.257	2252.886	− 0.136	0.892
Age:Dimension:Latency	0.950	0.952	2252.886	0.998	0.319
Age:Dimension:Direction	0.866	0.952	2253.205	0.909	0.364
Age:Latency:Direction	0.512	0.952	2252.886	0.538	0.591
Dimension:Latency:Direction	− 1.098	19.257	2252.886	− 0.057	0.955
Age:Dimension:Latency:Direction	0.455	0.952	2252.886	0.478	0.633

## 4 Discussion

The present study sought to determine at what age in perinatal development, infants start to reliably discriminate between native vowels. Prior studies show that infants learn about the native language already in utero, being able to recognize previously exposed language sound patterns such as the language itself, its melody, rhythm, and very likely also individual speech segments or syllables (Moon et al., 2013, Partanen et al., 2013). At the same time, newborn infants process language stimuli differently from non-speech signals (May et al., 2018, Chládková et al., 2021). The early neural processing of speech thus very likely develops differently than the early neural processing of non-speech sounds. Yet, a fine-grained trajectory of early – prenatal or postnatal – development of auditory perception has almost exclusively been assessed with non-speech stimuli (Rotteveel et al., 1987, Kushnerenko et al., 2002, Bisiacchi et al., 2009, Lippé et al., 2009, Suppiej et al., 2010). Studies that did use speech stimuli mostly compared preterm and fullterm infants at term age, thus not allowing to trace the very trajectory of the perinatal ERP development (François et al., 2021, Pena et al., 2012; Kostilainen et al., 2020). The aim of the present experiment was to test at which age the brain starts to distinguish between acoustically different vowels (all of which belong to the phoneme inventory of the infants' native language). To this end, we recruited newborns in the age range between 32 and 42 weeks of gestation (all tested a few days after birth) and measured their event-related potentials, as well as their auditory neural mismatch response, to native vowels differing in spectral quality and native vowels differing in duration.

A total of 99 sleeping newborns were played naturally produced, isolated vowels embedded in an oddball paradigm, half of the infants was tested with the vowels [ɛ] and [a] and the other half with the vowels [ɛ] and [ɛː]. Each infant listened to two oddball blocks, such that each of the two vowels served as a standard in one block and as a deviant in the other block. All these vowels represent phonemes in the infants' mothers' native language, Czech. We included both a spectral-change contrast (represented by the [ɛ] and [a]) as well as duration-change contrast (represented by the [ɛ] and [ɛː]) as we predicted that neural sensitivity might develop slightly earlier for the durational than for the spectral contrast. This prediction is based on the cue-specific properties of prenatal input, where durational cues are preserved in utero in an unchanged form, while spectral cues are modulated as they pass through tissues and amniotic water to the fetal ear (Richards et al., 1992; Granier-Deferre et al., 2011). Moreover, developmental studies with Czech-learning infants suggest more robust discrimination of vowel length compared to vowel quality across the first year of life, as well as exaggeration of durationally cued vowel contrasts in the infants' (prenatal) input (Chládková et al., 2021, Paillereau et al., 2021, Chládková et al., 2019, Svoboda et al., 2023). To assess the newborns' sensory processing of vowel acoustic properties we analysed the event-related potentials to vowel onsets and vowel offsets. To quantify the brain's neural discrimination of the vowel differences, we assessed the mismatch responses to the spectral change and to the durational change.

For the sensory responses to vowel onsets, the present analyses detected a positive peak from day 253 of gestation (at time of experiment), that is, from 36 weeks and 1 day. This finding is in line with the literature demonstrating that in newborns the most prominent auditory ERP is a positive peak slightly after 200 ms, which with age develops into a negative N1 peak as the prominent auditory ERP response, maturing into the P1-N1-P2 complex (Picton and Taylor, 2007; Wunderlich et al., 2006). The present ERP results showed that the infants' onset ERP responses reflected the spectral differences between [ɛ] and [a] from the age of 258 days (i.e., 36 weeks and 6 days), and their offset ERP responses reflected the durational difference between [ɛ] and [ɛː] from the age of 265 days (i.e. 37 weeks and 6 days). The onset response was more robust overall, which aligns with prior studies on adults that offset ERPs are smaller than onset ERPs (Baltzell and Billings, 2014). A previous study comparing auditory onset and offset responses in young infants suggests that a large offset response may be a marker of immature development (Wakai et al., 2007). The present study adds to that by showing that compared to onset responses, offset ERPs begin to reflect acoustic differences between vowels at a slightly later age, namely, one week later than onset ERPs. In the present study, onset responses reflected processing of the vowels' spectral characteristics and offset responses reflected processing of the vowels' durational characteristics. This might possibly indicate that the processing of spectral vowel contrasts matures earlier than the processing of vowel duration contrasts. However, in order to make conclusions about the order of maturation for the two types of contrasts, one would need to test them in a single model, ideally using a within-subject design, and – as far as possible – unconfounded by the positional context (i.e. whether the vowel change occurs at stimulus onset vs offset).

The MMR data showed that the newborns' brains discriminated the change in vowel duration, i.e. discriminated the speech contrast represented by [ɛ]–[ɛː], while no evidence of discrimination was found for vowel spectral quality. Interestingly, the MMR polarity inversely correlated with age, it was positive in the youngest infants and negative for the oldest infants. Although this developmental polarity change would align with some prior studies claiming a developmentally-conditioned MMR polarity (see Govaart et al., 2023 for a review), it is questionable to what extent one can validly assess an MMR response in the absence of reliably different sensory ERPs (Kremláček et al., 2016). We thus make no further inferences here regarding the MMR detected in infants younger than 253 days, since it was from this age when the vowel stimuli elicited a sensory ERP reliably different from zero. Considering the newborns older than 253 days, an MMR reliably different from 0, here with negative polarity, was elicited from the age of 285 days, that is, 40 weeks and 4 days. The presence of an MMR for the durational contrast (and the failure to detect it for the spectral contrast) aligns with prior studies documenting greater perceptual sensitivity in Czech-learning infants to vowel duration changes than to vowel spectral changes (Chládková et al., 2021, Paillereau et al., 2021).

The question remains whether the early maturation of an MMR response to vowel duration specifically (and its lack for vowel spectral changes) is language-specific, dependent on the infants' early prenatal input, or whether it is a language-general property of the developing speech perception system. To this end, a comparison to an earlier study with Finnish-learning newborns seems to speak in favour of language-specific MMR patterns at birth as the study with Finnish newborns found an MMR both for durational and spectral vowel contrasts, and detected an MMR in both an early and a late time window. This is, however, only a very rough comparison, since the two studies used very different types of stimuli (isolated vowels here and disyllabic words in the Finnish study) and different recording procedures and analysis pipelines. Note that the present study was not designed to test language-specific vs language-universal newborn speech perception and the present results cannot be interpreted in terms of language-specific phonological category learning in the perinatal period. The present results track the perinatal development of neural processing of different types of vowels, all of which happen to be realisations of phonemes in the ambient language.

The present findings contribute a more detailed understanding of the developmental trajectory of speech perception development in the perinatal period. Firstly, the present finding that reliable onset ERPs were elicited from gestational age (at time of eperiment) of 36 weeks and 1 day aligns well with the maturation of auditory brainstem potentials that were reported to stabilise at gestation week 36 (Starr et al., 1977). Secondly, as to the differentiation of acoustically distinct speech sounds, we identified 36 weeks and 6 days as the age from which the cortex of (Czech-learning) newborns differentiates (at least some of) the native vowel identities. Since the ERPs are locked to the very occurrence of the target phonetic property, the present findings demonstrate a temporally rather precise phonetic perception three weeks prior to term age. Note that pevious research has indicated that the newborn cortex is able to differentiate between strings of [ga] and [ba] syllables already before 35 weeks of gestation (assessing blood oxygenation levels in Mahmoudzadeh et al., 2013, and ERPs in Mahmoudzadeh et al., 2017). Compared to Mahmoudzadeh et al. (2017), the later onset of reliable ERPs in our experiment might be due to different stimulus identities and presentation paradigms (blocks of syllables interspersed by silences in the previous study vs trains of vowels in the present study), different intensity levels (70 dB in the previous study vs 65 dB in the present study), or different procedures and equipment. The present findings of precise phonetic perception just prior to term age extend our knowledge on the capacities of the newborn brain to learn the ambient speech sounds. Using EEG and fNIRS, previous studies documented fast phonetic learning for previously unexposed vowels in full-term newborns and two-month old infants (Cheour et al., 2002, Wanrooij et al., 2014, Wu et al., 2022). Our findings of reliably differentiated ERPs from the age of 36 weeks and 6 days demonstrate that accurate phonetic perception of contrastive vowel properties is in place before (or at least at the same time as) the age at which infants have been reported to learn novel vowels from exposure.

The age at which ERPs start to reliably distinguish between acoustically different vowels seems to rather well coincide with the age that is, in many countries considered as the term age. In that respect, ERPs to vowel onsets and offsets might have the potential to help identify children with developmental delays, particularly those pertaining to speech and language, such as dyslexia. Hämäläinen et al. (2013) showed that atypical ERPs to speech and nonspeech sounds in preschool children are related to poorer reading abilities at school age. Atypical speech processing seems to index dyslexia already at birth in that full term newborns with familial risk of dyslexia reportedly showed delayed, attenuated, or even lacking MMR responses to vowel changes in disyllabic words (Thiede et al., 2019). Since the occurrence of primary ERPs reliably distinguishing phonetically different vowels coincides here quite well with term age, i.e. the age of maturation, recording the primary ERPs to isolated vowel sounds might prove as a suitable method for assessing developmental language delays at birth. Future research is needed to collect normative data on ERPs to isolated speech sounds at birth (for the language community at hand) and test whether newborns whose ERPs deviate from the norm develop speech or language pathologies later in life. If that is the case, the early identification of potential language delays would allow targeting a focused therapy (more speech input, more systematic input. etc.) from the earliest possible age.

## CRediT authorship contribution statement

**Jan Kremláček:** Writing – review & editing, Writing – original draft, Visualization, Supervision, Software, Resources, Methodology, Funding acquisition, Formal analysis, Data curation, Conceptualization. **Kateřina Chládková:** Writing – review & editing, Writing – original draft, Visualization, Supervision, Software, Resources, Project administration, Methodology, Funding acquisition, Formal analysis, Data curation, Conceptualization. **Josef Urbanec:** Writing – review & editing, Writing – original draft, Visualization, Resources, Project administration, Methodology, Investigation, Conceptualization.

## Declaration of Competing Interest

The authors declare that they have no known competing financial interests or personal relationships that could have appeared to influence the work reported in this paper.

## Data Availability

The data accompanying the present manuscript are available at https://osf.io/84czw/?view_only=3c355a0c8db6482297b145e221fc4503 (measured ERP and MMR amplitudes per infant and channel, as well as anonymized infant characteristics). Raw EEG data are available from the corresponding author upon reasonable request.
